# Sessile serrated lesion presenting as large pedunculated polyp in the rectum: A case report

**DOI:** 10.1097/MD.0000000000032287

**Published:** 2022-12-23

**Authors:** Shin Ju Oh, Jung-Wook Kim, Chi Hyuk Oh

**Affiliations:** a Division of Gastroenterology and Hepatology, Department of Internal Medicine, Kyung Hee University College of Medicine, Seoul, Korea.

**Keywords:** pedunculated polyp, rectum, serrated polyp

## Abstract

Sessile serrated lesions (SSLs) are serrated polyps (SP) with the typical serrated architecture of the crypt lining epithelium. SSL has an important clinical implication because they are recognized as precursor lesion of sporadic colorectal cancer (CRC) through “serrated pathway.” SSLs usually appear flat to sessile, and are located in the right colon.

**Patient concerns:**

A 69-year-old man was referred to a tertiary medical center because of intermittent hematochezia for 2 years.

**Diagnosis:**

Colonoscopy revealed a large, pedunculated polyp in the rectum. The polyp surface was slightly reddish in color and the elongated stalk was covered with almost normal mucosa. Histopathological examination of the resected specimens revealed the typical features of SSL with low-grade dysplasia.

**Intervention:**

Endoscopic mucosal resection using a detachable snare was performed on the tumor for definite diagnosis and treatment.

**Outcomes:**

There was no evidence of immediate or delayed bleeding after endoscopic mucosal resection, and the hemoglobin level normalized after a 1-year follow-up.

**Lessons:**

We report a rare case of a large pedunculated polyp with typical histological features of SSLs in the rectum. Endoscopists should always consider SSLs at any location even with unusual morphological findings.

## 1. Introduction

In 1990, Longacre and Fenoglio-Preiser first described polyps characterized by a serrated architecture and unequivocal dysplasia known as sessile serrated adenoma/polyp (SSA/P).^[1]^ Recently, the sessile serrated lesion (SSL) is a new terminology that replace SSA/P according to the 2019 World Health Organization (WHO) classification.^[2]^ SSLs are clinically significant because they are potential precursors of sporadic microsatellite unstable colorectal cancer (CRC), accounting for 15% to 30% of all CRCs.^[3,4]^ Somatic BRAF mutations and DNA CpG island hypermethylation (CIMP) are the key molecular features of the serrated pathway.^[5,6]^

Morphologically, SSLs are usually flat to sessile and similar in color to the surrounding mucosa. Under endoscopy, the presence of a mucus cap, rim of bubbles or debris, alteration of the contour of the mucosal fold, and/or loss of the normal mucosal vascular pattern also suggest the possibility of SSLs.^[7]^ These lesions are predominantly found in the proximal colon.^[8]^ As rare forms of SSLs, pedunculated polyps or depressed surfaces have also been described.^[9,10]^ Most SSLs with these features were also located in the right-sided colon and small in size. Herein, we report a large pedunculated polyp with the typical histological features of SSLs in the rectum.

## 2. Case presentation

A 69-year-old man was referred to our clinic with intermittent, scanty hematochezia and dizziness that had persisted for 2 years. He had no medical history and had never undergone a colonoscopy. The patient complained of a mass in his anus that had spontaneously reduced. No signs of hemodynamic instability were observed during outpatient clinic visits. The vital signs of the patient were as follows: blood pressure, 122/53 mm Hg; pulse rate, 82 beats per minute; and body temperature, 36.9°C. A physical examination revealed no specific findings. The initial laboratory data showed a hemoglobin concentration of 3.7 g/dL (normal range:12.0–16.0).

**Figure 1.**
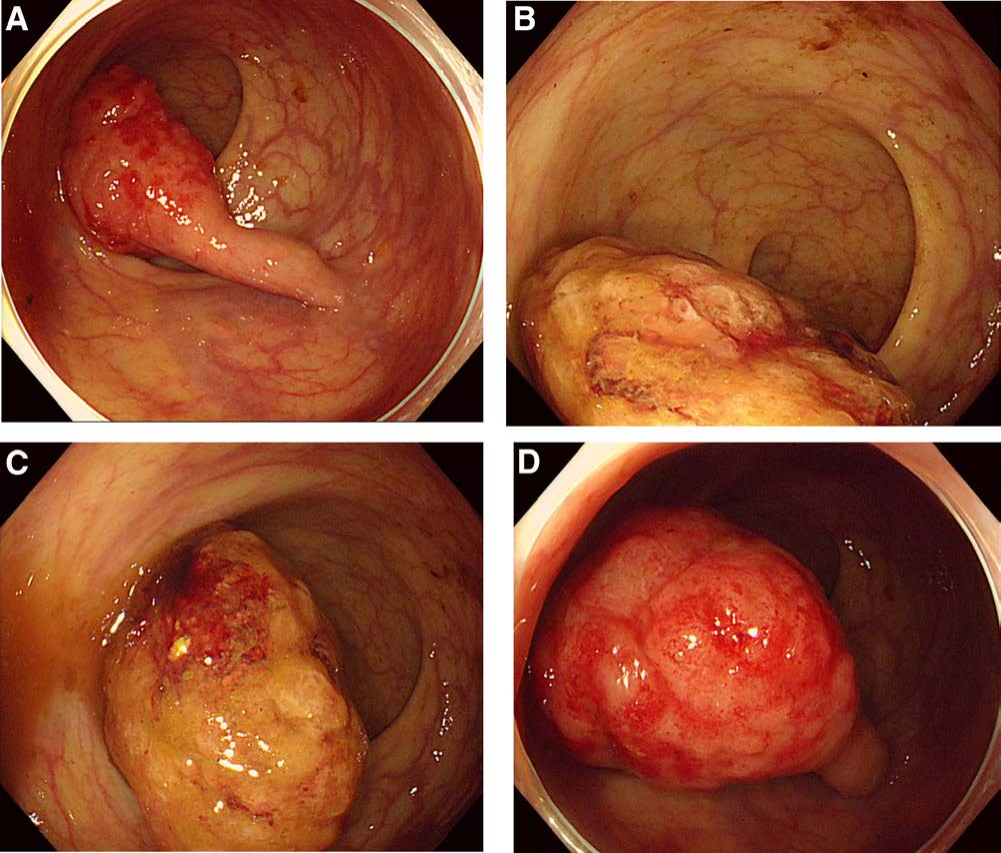
(a-d) Endoscopic finding of a large pedunculated polyp in the rectum.

**Figure 2.**
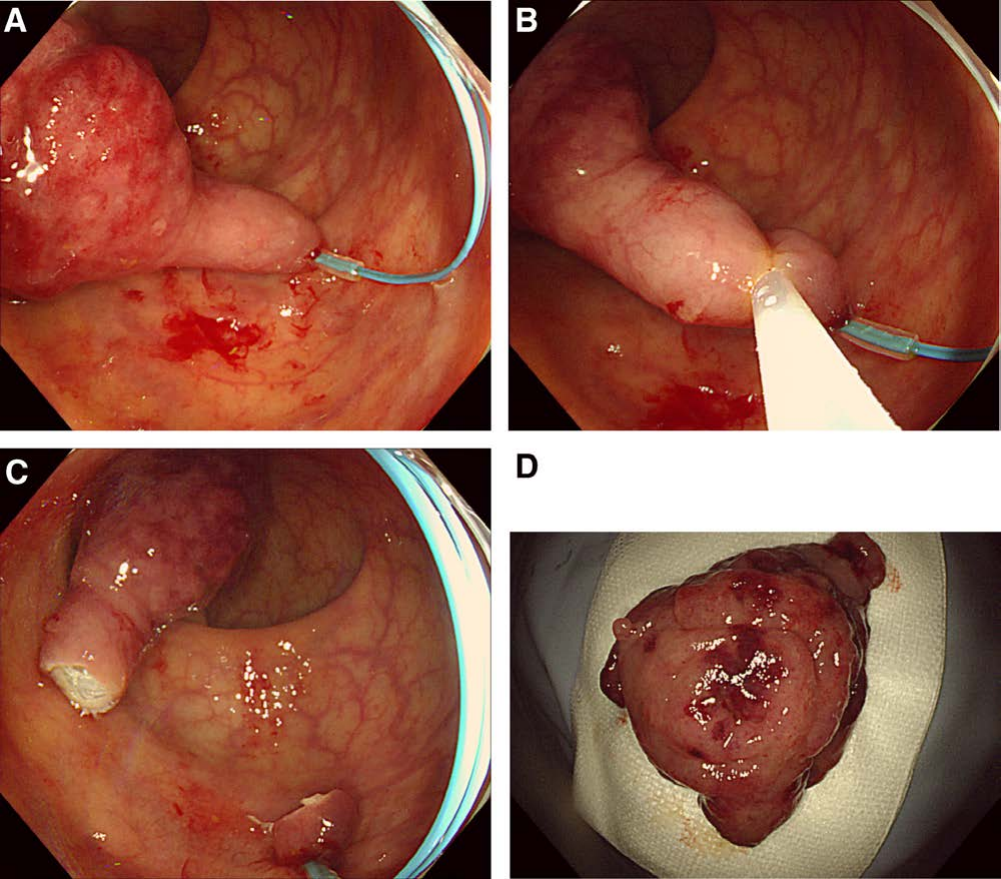
Procedure of endoscopic mucosal resection (EMR). (a) The base of the polyp was ligated with Endoloop. (b and c) Then, EMR was performed using a 25-mm snare, and the polyp was completely resected. (d) Macroscopic appearance of the resected polyp.

Initially, colonoscopy was performed to identify the cause of anemia and hematochezia. Colonoscopy revealed a large, pedunculated polyp in the rectum. However, we could not find any colorectal polyps other than rectal lesions during initial colonoscopy. The surface of the polyp was slightly reddish in color and was surrounded by mucus without a peripheral rim of debris or bubbles. The elongated stalk was covered with almost normal mucosa (Fig. 1). A biopsy specimen was obtained from the reddish part of the surface; however, histological examination revealed chronic inflammation without neoplastic changes. However, the polyp was suspected to be a neoplastic lesion based on the endoscopic features, including size, so we decided to perform endoscopic resection of the tumor for definite diagnosis and treatment. After submucosal injection of normal saline, a detachable snare (Endoloop, MAJ-254; Olympus Corp., Tokyo, Japan) was placed on the base of the stalk to prevent immediate bleeding. The polyp using a 25-mm snare (SD-221U-25; Olympus Corp., Tokyo, Japan) without any other complications (Fig. 2).

**Figure 3.**
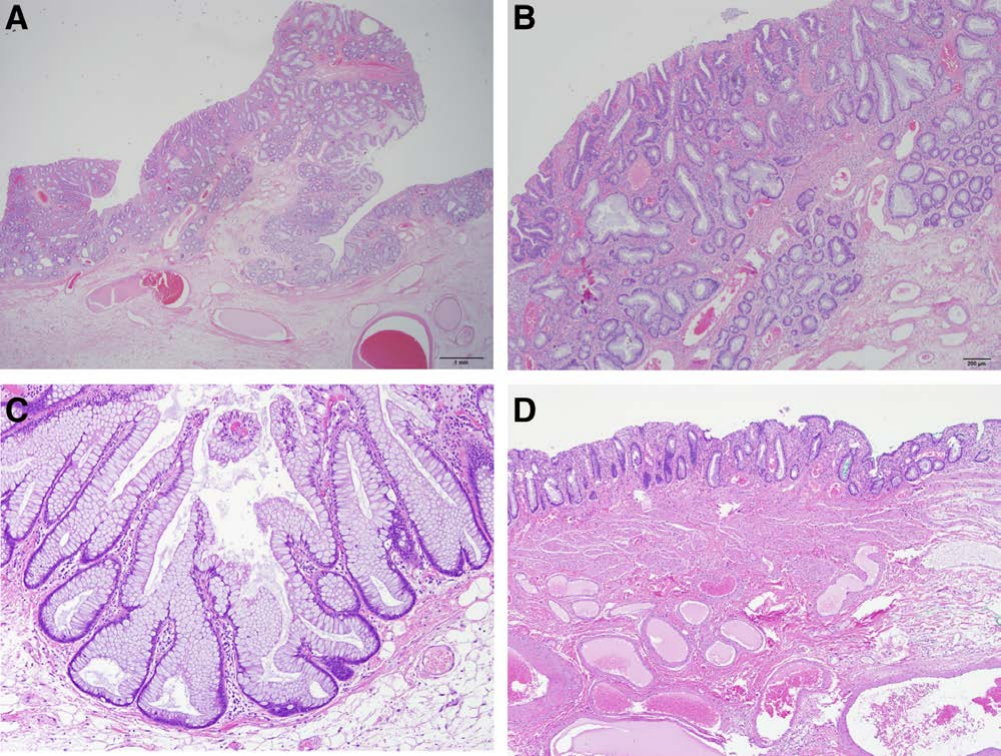
Histopathologic findings. (a) Low-power view of a pedunculated polyp which included adenomatous epithelium in about 10% of the polyp structure. (b) Cytologic dysplasia and nuclear hyperchromasia and elongation were noted. (c) The polyp shows inverted T-shaped or L-shaped crypts with abundant luminal mucin d. The stalk consists of loose connective tissue without dysplasia.

The pathologic result was a pedunculated serrated lesion with low-grade dysplasia 4.0 × 4.0 × 2.0 cm in size, which included adenomatous epithelium in about 10% of the polyp structure (Fig. 3a and b). Histopathological examination showed the typical features of serrated lesions, such as dilatation and branching of the basal crypts and inverted T- or L-shaped crypts with abundant luminal mucin (Fig. 3c). However, there were no diamond-shaped crypts or smooth muscle proliferation in the lamina propria, which is characteristic of hyperplastic polyps (HP) with prolapse.^[11]^ And no histological evidence of cytoplasmic eosinophilia or ectopic crypt formation was observed. Thus, we were able to differentiate the lesion from the TSA.^[9]^ The stalk of the polyp consisted of loose connective tissue within the submucosal layer (Fig. 3d). To assess BRAF mutation status, genomic DNA was extracted, and BRAF mutations were analyzed using the real-time PCR clamping technology of the PNAClampTM BRAF kit; BRAF mutations were not detected in this case.

There was no evidence of immediate or delayed bleeding after endoscopic mucosal resection, and hemoglobin level was normalized (Hgb 13.4 g/dL) up to the year follow-up.

## 3. Ethic statement

The requirement for institutional review board approval was waived owing to the retrospective nature of the study. Written informed consent was obtained from the patient and his legal guardian for the publication of this article and any accompanying images.

## 4. Discussion

The 2019 WHO recognized 4 categories of serrated colorectal lesions: HP, SSL, SSLs with dysplasia, traditional serrated adenomas, and unclassified adenomas.^[2]^ Serrated lesions with abnormal proliferation are characterized by horizontally growing crypts along the muscularis mucosa, a dilated crypt base, serrated morphology throughout the crypt, including the base, and asymmetrical proliferative features of the crypt. This is caused by the migration of the proliferative zone to the side of the crypt, which is a distinguishing feature of HPs.^[3]^ SSLs are clinically important because, as recent research has indicated, they are the precursor lesions of the serrated pathway to CRC.

BRAF is a member of the RAF family of serine/threonine-protein kinases. As a result of the V600E mutation, BRAF and its pathway are activated and are strongly associated with CIMP-positive cancers. BRAF mutations are reported to be present in 78% to 100% of SSLs and 0% to 0.4% of tubular adenomas, supporting the importance of SSLs as precursors of CRC.^[12]^ Kim et al reported a pedunculated polyp with histopathological features of SSLs, and all pedunculated polyps showed a BRAF-V600E mutation.^[9]^ Previous studies have suggested the possibility of a relationship between BRAF mutations and apoptosis; thus, it may play an important role in the developmental process of flat and depressed morphology.^[13]^ In this case, wild-type BRAF may be related to pedunculated morphology, although this has not yet been well established. Furthermore, it is possible that the lesion is KRAS-associated serrated, although KRAS mutations have been identified in approximately 6% of Asian patients.^[14]^ Unfortunately, we were unable to confirm the KRAS mutation in this case.

The prevalence of SSLs is approximately 25% of all serrated polyps (SP), and ranges from 2% to 8% in average-risk patients undergoing colonoscopy.^[4,15,16]^ However, more recent study presented high prevalence (up to 15%) of SSA/Ps in a screening population by high-detecting endoscopist and an experienced pathologist.^[17]^ SSLs usually appear flat or sessile endoscopically; 98.1% of lesions are flat according to the Paris classification,^[7]^ and 75% to 90% are right-sided.^[18]^ In addition, they have soft and smooth surfaces and are often covered with mucus caps. However, the morphological characteristics of SSLs remain poorly characterized. Recent studies have suggested an unusual form of SSLs within the spectrum of serrated neoplasia.^[9,10]^ Kim et al described 5 pedunculated SP with histological features of SSLs. These polyps showed a pedunculated configuration consisting of a small globular head and short and weak pedicle.^[9]^ All polyps were proximally located and were less than 1 cm in size. Another study described SSLs with a depressed surface, which, according to the Paris classification, corresponds to type 0-IIa + IIc or 0-Is + IIc.^[10]^ Colonic polyps rarely cause symptoms and are usually incidentally detected. Furthermore, previous studies have suggested that SSLs may cause fewer symptoms than adenomas, making them difficult to detect clinically.^[19]^ Despite a pathologically confirmed SSL, this is a rare case of hematochezia and anemia owing to its large size and morphology.

In the present case, a large pedunculated polyp with a long, thick stalk was found in the rectum. Unlike the previously reported pedunculated forms of SSLs, this polyp could be misdiagnosed as a colon hamartoma or adenoma based on its morphological features and location. As previously suggested, this lesion can be a rare variant of SSLs or a precursor or result of an SSL.^[7]^ Further studies of the endoscopic morphology and biological nature of these lesions are required.

In conclusion, we report an unusual case of large pedunculated SSL in the rectum. The polyp was treated with endoscopic mucosal resection and pathologically confirmed as an SSL. Our findings indicate that endoscopists should always keep in mind SSLs in any location, even with unusual morphological findings.

## Acknowledgments

We sincerely express our gratitude to Prof Ji-Youn Sung (MD, PhD) from the Department of Pathology, Kyung Hee University School of Medicine.

## Author contributions

SJO, JWK, and CHO designed and drafted the manuscript, and substantively revised the manuscript. All authors approved the submitted version (and any substantially modified version that involved the author’s contribution to the study). All authors agreed to be personally accountable for the author’s own contributions and to ensure that questions related to the accuracy or integrity of any part of the work, even those in which the author was not personally involved, were appropriately investigated and resolved, as documented in the literature.

**Conceptualization:** Shin Ju Oh, Jung-Wook Kim, Chi Hyuk Oh.

**Writing – original draft:** Shin Ju Oh, Jung-Wook Kim, Chi Hyuk Oh.

**Writing – review & editing:** Shin Ju Oh, Jung-Wook Kim, Chi Hyuk Oh.
